# ^2^H Solid-State NMR Analysis of the Dynamics and Organization of Water in Hydrated Chitosan

**DOI:** 10.3390/polym8040149

**Published:** 2016-04-19

**Authors:** Fenfen Wang, Rongchun Zhang, Tiehong Chen, Pingchuan Sun

**Affiliations:** 1 Key Laboratory of Functional Polymer Materials of Ministry of Education and College of Chemistry; Nankai University, Tianjin 300071, China; wff@mail.nankai.edu.cn (F.W.); chenth@nankai.edu.cn (T.C.); 2 State Key Laboratory of Medicinal Chemical Biology, Nankai University, Tianjin 300071, China; 3 Biophysics and Department of Chemistry, University of Michigan, Ann Arbor, MI 48109-1055, USA; zrcrong@gmail.com; 4 Collaborative Innovation Center of Chemical Science and Engineering (Tianjin), Nankai University, Tianjin 300071, China

**Keywords:** water state, hydrated chitosan films, mobility, ^2^H solid-state NMR, variable-temperature

## Abstract

Understanding water–biopolymer interactions, which strongly affect the function and properties of biopolymer-based tissue engineering and drug delivery materials, remains a challenge. Chitosan, which is an important biopolymer for the construction of artificial tissue grafts and for drug delivery, has attracted extensive attention in recent decades, where neutralization with an alkali solution can substantially enhance the final properties of chitosan films cast from an acidic solution. In this work, to elucidate the effect of water on the properties of chitosan films, we investigated the dynamics and different states of water in non-neutralized (CTS-A) and neutralized (CTS-N) hydrated chitosan by mobility selective variable-temperature (VT) ^2^H solid-state NMR spectroscopy. Four distinct types of water exist in all of the samples with regards to dynamic behavior. First, non-freezable, rigid and strongly bound water was found in the crystalline domain at low temperatures. The second component consists of weakly bound water, which is highly mobile and exhibits isotropic motion, even below 260 K. Another type of water undergoes well-defined 180° flips around their bisector axis. Moreover, free water is also present in the films. For the CTS-A sample in particular, another special water species were bounded to acetic acid molecules via strong hydrogen bonding. In the case of CTS-N, the onset of motions of the weakly bound water molecules at 260 K was revealed by ^2^H-NMR spectroscopy. This water is not crystalline, even below 260 K, which is also the major contribution to the flexibility of chitosan chains and thus toughness of materials. By contrast, such motion was not observed in CTS-A. On the basis of the ^2^H solid-state NMR results, it is concluded that the unique toughness of CTS-N mainly originates from the weakly bound water as well as the interactions between water and the chitosan chains.

## 1. Introduction

Water plays a key role in controlling the structure and function of biomacromolecules in nature, such as switching of cell channels, directing protein folding, stabilizing structures, *etc*. [1,2]. In particular, nature uses an intriguing strategy to make high-performance and degradable biomaterials via subtle control of the interactions between water and biomacromolecules. One example is spider silk, which exhibits unparalleled properties of stiffness, tensile strength, and toughness due to exposure to water during stretching as well as a unique supercontraction phenomenon upon absorption of water [3]. Biopolymers are the best choice for the preparation of structural materials, such as scaffolds and replica bone due to their outstanding biodegradability and biocompatibility [4,5]. However, the properties of polymer materials are often strongly affected by the polymer–water interactions, the understanding of which remains one of the great challenges in modern macromolecular science, especially with respect to the dynamics and organization of water in the polymer matrix.

Chitosan, which is an *N*-deacetylated derivative of chitin, has been widely used in the field of tissue engineering [4,6,7,8]. Chitosan and other polysaccharides tend to adsorb water at high atmospheric humidity, which can drastically modulate the dynamics and mechanical properties of these biopolymers [9]. Therefore, in general, the mechanical properties of polysaccharides or polysaccharide-containing materials strongly depend on their water content, even if it is very low. As reported in the literature, the water absorbed in biopolymer systems can exist in three states: non-freezable water, freezable bound water (weakly bound water), and free water [10]. In particular, variation of the location of bound water molecules will induce considerable changes to the physicochemical properties of polymeric materials. Therefore, the locations of bound water molecules as well as the interactions among water and polymers molecules need to be well determined and understood. Moreover, the mobility of the absorbed water in hydrogels is important for their use in sustained release, tissue engineering, and other biomedical and biotechnological applications [11,12,13]. Therefore, the mobility of water in polymer hydrogel materials must be studied.

Water molecules often act as a plasticizer in the polymers, rendering the biomaterials softer. However, elucidating the molecular mechanism responsible for the interactions between water and biopolymers still remains a challenge. In the past, dielectric spectroscopy, infrared analysis neutron reflectivity and thermal analysis have been widely used to investigate water-polymer interactions [14,15,16]. In particular, differential scanning calorimetry (DSC) is a useful technique that is very sensitive to thermal transitions in multiphase systems and has been used to investigate the different states of water in hydrogels [17]. However, DSC is not sensitive at all when there is only a very small amount of water in biopolymer materials.

Solid-state NMR (SSNMR) spectroscopy is a nondestructive and powerful technique for studying the structure and dynamics of biopolymers as well as the water–biopolymer interactions [18,19,20]. In a previous study, ^17^O NMR was used to gain structural insights into the bound water in crystalline amino acids [21]. However, the low natural abundance (0.037%), small gyromagnetic ratio, and quadrupolar nature of the ^17^O nuclei make the detection of the states and dynamics of trace water in biopolymers almost impossible if hyperpolarization method was not used. Fortunately, the ^2^H solid-state NMR technique is a powerful tool for exploring the molecular-level structures and dynamics of water in hydrated systems [22,23,24,25]. For example, ^2^H NMR has been employed to characterize the local mobility of two different types of water molecules in a synthetic water channel [23], to study the behavior of D_2_O in hydrated nylon-6 fibers [26], and to investigate the water-polymer interactions in hydrated biomacromolecule materials [19,27]. Herein, we used ^2^H SSNMR to investigate the dynamics and different states of water in non-neutralized (CTS-A) and neutralized (CTS-N) hydrated chitosan films as well as the relationship between the states of water and the mechanical properties of chitosan films. To distinguish the different states of water associated with different molecular mobility and their temperature dependence, mobility selective variable-temperature (VT) ^2^H NMR was carried out from 190 to 320 K. Moreover, the temperature dependence of weakly bound water was determined for both samples in this study, which plays a key role in determining the toughness of chitosan materials.

## 2. Experimental Section

### 2.1. Materials

Chitosan powder was purchased from Sigma-Aldrich (Shanghai, China) (high molecular weight, *M*η = 700,000 (by viscometric method) [28]); its degree of deacetylation was determined to be 86.3% using NMR spectroscopy as reported before [29]. D_2_O, NaOH and CH_3_COOH were analytically pure.

### 2.2. Preparation of Chitosan Films

Chitosan powder was dissolved in a 2 *w*/*v* % acetic acid solution with subsequent stirring to promote dissolution. The films were cast from these solutions and dried at 318 K under vacuum (named CTS-A); these films still contain residual acid. The dried films were then neutralized by immersion into a 4 *w*/*v* % solution of NaOH followed by adequate washing with distilled water and drying at 318 K under vacuum (named CTS-N). These two samples were immersed in D_2_O (99.9%) for 2 h and then dried at 318 K under vacuum. The resulting water content was approximately 8 wt % in both samples (*i.e.*, CTS-A and CTS-N) as determined by the thermogravimetric analysis (TGA) experiments (see Figure S2 for the thermogravimetric analysis in Supplementary Materials). All of the samples were sealed in glass tubes in order to keep the water content. Notably, the chemical exchange between D_2_O and the chitosan –OH and –NH groups also occurred at room temperature. This type of ^2^H signal should be taken into account for interpretation of the spectra presented in this work. CTS-N films for tensile measurement were prepared by first immersed the films into water for 1 h, then taking the films out and dried at room temperature for 0, 1, and 2 h with the water content of 71.4%, 45%, and 28.2% determined by TGA (not included here), respectively.

### 2.3. Tensile Measurements

Tensile stress-strain tests were performed at room temperature on a Testometric AX universal strength testing machine (Testometric Co. Ltd., Manchester, UK) using a 50 mm/min strain rate. The dumbbell-shaped specimens were stamped from the chitosan films using gauge dimensions of 12 × 2 mm^2^ with a thickness of 0.3–0.4 mm, which was measured using an SFJ digital thickness tester for each sample. Each data point was the average from measurements of at least five samples.

### 2.4. X-ray Diffraction (XRD)

The XRD experiments were performed in reflection mode on a D/max-2500 X-ray powder diffractometer (Rigaku Corporation, Tokyo, Japan) equipped with a Cu Kα (λ = 0.154 nm) radiation source operated at 40 kV and 100 mA.

### 2.5. Solid-State NMR Experiments

The NMR experiments were performed at room temperature (25 °C) on a Varian Infinitplus-400 wide-bore (89 mm) NMR spectrometer (Agilent Technologies Inc., Santa Clara, CA, USA) at a frequency of 61.21 MHz for ^2^H. A HX CP/MAS probe with a rotor diameter of 4 mm was used, and samples with a volume of 52 μL were placed in a zirconia PENCIL rotor (Agilent Technologies Inc., Santa Clara, CA, USA). The variable-temperature experiments were carried out in the temperature range from 190 to 320 K using a Varian model L950 temperature controller. The temperature was allowed to equilibrate for 30 min at each temperature before the NMR spectra were recorded. For all of the *T*_1_-selective ^2^H experiments, three different pulse sequences were used. A quadrupolar echo (90°-τ-90°-τ-acquire) sequence was used to obtain a undistorted spectrum, which corresponds to all of the ^2^H signal species in the hydrated chitosan. To selectively excite the fast-relaxing species and distinguish different types of water, a series of saturation pulses were incorporated into the quadrupolar echo sequence ((90°)_n-times_-τ_1_-90°-τ_2_-90°-τ_2_-acquire, where a short τ_1_ selects only the fast-relaxing (*i.e.*, mobile) component. For all the experiments applied in this study, *n* = 20 and τ_1_ = 10 ms was applied [15]. The 90° pulse length was approximately 2.9 μs, and the pulse delay of the solid echo sequence (τ and τ_2_) was set to 40 μs. Furthermore, a Torchia’s T_1_ [30] was incorporated into the quadrupolar echo sequence [30] (90°-τ_2_-90°-τ_2_-90°-τ_T1_-90°-acquire, named Torchia’s T_1_-filter quadrupolar echo here) to selectively acquire the ^2^H signal of rigid/crystalline water at low temperatures. Actually, the spectrum of mobile components could also be obtained by the differential of two spectra obtained with τ_T1_ set to 0 and 2.5 s, respectively.

## 3. Results and Discussion

### 3.1. Crystallinity and Mechanical Properties of Hydrated Chitosan Films Based on XRD and Tensile Experiments

**Figure 1:**
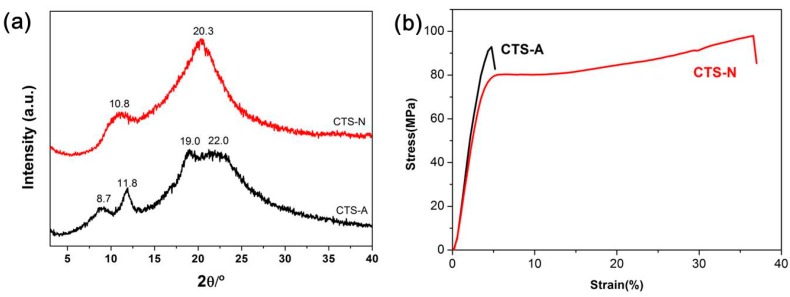
(**a**) XRD patterns of CTS-A and CTS-N at room temperature; and (**b**) stress–strain curves of CTS-A and CTS-N.

The crystallization behavior of the CTS-A and CTS-N films was characterized using XRD, as shown in Figure 1a. The X-ray powder pattern of chitosan in Figure 1 is consistent with the previously reported results [31]. The peaks at 2θ = 8.7°, 11.3° and 22.0° are the characteristic diffraction peaks of chitosan acetic salt, and the peaks at 2θ = 10.8° and 20.3° correspond to the equatorial (100) and (020) reflections, respectively, of neutralized chitosan. The degree of crystallinity was 14.8% for CTS-A and 25.8% for CTS-N on the basis of the peak-differentiation-imitating analysis of the two 1D profiles (see Figure S3 in the Supplementary Materials). The mechanical properties of the two CTS films were investigated using tensile measurements at room temperature, and the obtained stress–strain curves are shown in Figure 1b. The derived mechanical parameters, including the Young’s modulus, stress and elongation at break, are summarized in Table 1. Apparently, CTS-N exhibited a ductile fracture with a large elongation of 35.8%, while the elongation of CTS-A was much smaller than that of CTS-N. In fact, such crystalline and mechanical difference could be ascribed to the different dynamics and organization of water in the two hydrated chitosan films, as discussed in detail below. The effect of the water content on the mechanical properties of CTS films is shown in Figure S1 and listed in Table S1. For CTS-N films at water content greater than 8 wt %, an obvious decrease of the mechanical properties can be observed because of the plasticization effect of water.

### 3.2. Dynamics and Different States of Water in Hydrated Chitosan before and after Neutralization Using Dynamic-Editing VT ^2^H NMR Experiments

^2^H NMR has been successfully used to reveal the orientational ordering and phase dynamics in a wide range of liquid crystals, polymers and biopolymers [32]. The ^2^H solid-state spectra are dominated by the quadrupole coupling of the deuteron spins. The splitting between the peaks in ^2^H spectra (Δ*ν_Q_*) is determined by the quadrupolar interactions,

(1) $$\Delta \nu_{Q}=\frac{3}{4}(\frac{e^{2}qQ}{h})(3\cos^{2}\theta (t)-1)$$

where *e*^2^*qQ*/*h* is the quadruple coupling constant and θ is the angle of the ^2^H–N or ^2^H–O bond relative to the magnetic field in the chitosan samples. A distribution of angles, which is present in polycrystalline or amorphous samples, generates the so-called Pake pattern [33]. The molecular motions will induce the averaging of quadrupolar couplings, leading to the collapse of the Pake pattern into a narrow isotropic peak. According to the value of peak splitting, multiple components could be separated and determined based on the dynamic differences.

**Figure 2:**
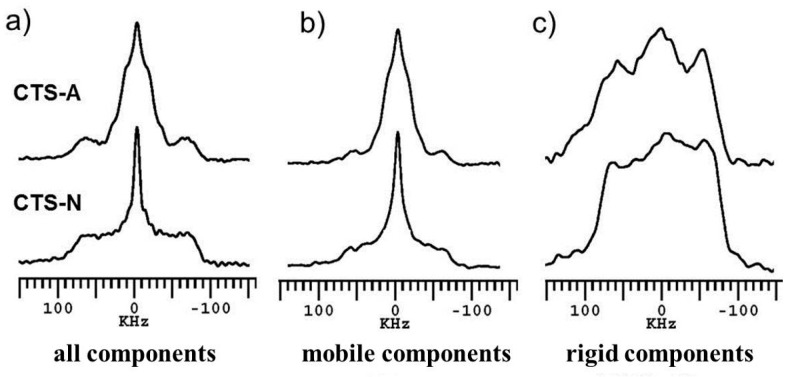
Dynamic-editing ^2^H NMR spectra of CTS-A and CTS-N at room temperature: (**a**) solid-echo spectra recorded with a repetition time of 2 s; (**b**) saturation Recovery quadrupolar echo spectra with a saturation recovery delay τ_1_ = 10 ms to selectively observe signals of mobile components; and (**c**) Torchia’s T_1_-filter spectra to selectively observe signals of rigid components.

The dynamic-editing ^2^H NMR spectra of CTS-A and CTS-N at room temperature are shown in Figure 2, where different states of water are observed. First, the solid-echo sequence was used to obtain the undistorted spectra containing the signals of all ^2^H spins, as shown in Figure 2a, where the heterogeneous dynamics of different water species were observed from the peak splitting patterns. All of the spectra shown in Figure 2a contain overlapping peaks with a narrowed line at the center of the spectra and a broad Pake pattern at the bottom. This result indicates that the ^2^H nuclei in the CTS samples exhibit heterogeneous dynamic behavior. The mobile components with narrow lines (Figure 2a) can be separately detected via the saturation echo experiment with short τ_1_, as shown in Figure 2b. The results in Figure 2b indicate that the ^2^H spins with fast motion contain two species as follows: a broad peak corresponding to –ND/–OD groups resulted from the chemical exchange between chitosan and D_2_O and a narrow peak corresponding to water molecules. Notably, the spectral width of the central narrow peak of CTS-A was much larger than that of CTS-N, indicating a distinct difference in the mobile water dynamics and organization between the two samples. In addition, the mobile water molecules in CTS-A are strongly bound to the chitosan chains and led to remarkable line broadening, which will be further confirmed by VT ^2^H NMR below. The ^2^H spectra of the slow-relaxing (rigid) components were separately determined by Torchia’s T_1_ filter quadrupolar echo experiments, as shown in Figure 2c. For CTS-A and CTS-N, the very broad peak indicates the presence of strongly bound water in the chitosan films. According to a previous study on cellulose using ^2^H NMR [24], the broad peaks shown in Figure 2c may arise from room-temperature chemical exchange between D_2_O and the –OH and –NH groups of chitosan, which leads to isotopic labeling of chitosan. In addition, one type of water molecule exists in the chitosan crystalline domain as reported before [34]. Therefore, the strongly bound water in the crystalline phase should also contribute to the broad signal. We have also taken the deacetylation degree and molecular weight of chitosan samples. As shown in Figure S4, the spectra shape were almost the same, so this two aspects were not influence the conclusion below.

**Figure 3:**
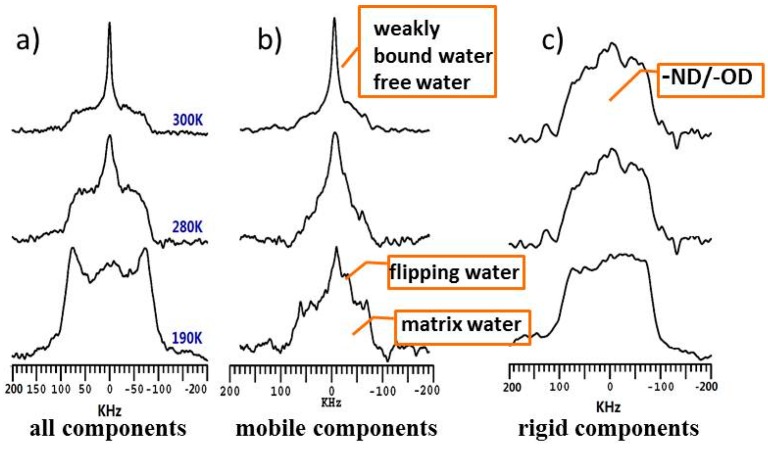
Dynamic-editing VT ^2^H NMR spectra for CTS-N obtained from: (**a**) quadrupolar echo; (**b**) saturation recovery quadrupolar echo; and (**c**) Torchia’s T_1_ filter quadrupolar echo experiments, respectively.

**Figure 4:**
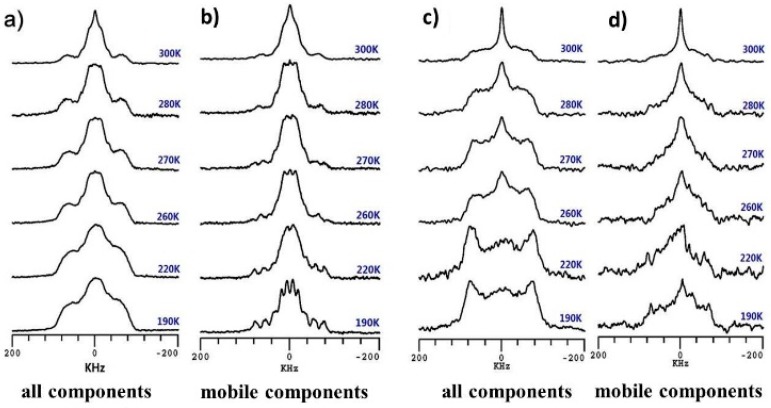
VT ^2^H spectra obtained from: (**a**,**c**) quadrupolar echo experiments to obtain signals of all components; and (**b**,**d**) saturation recovery quadrupolar echo experiments to obtain signals of mobile components for CTS-A (**a**,**b**) and CTS-N (**c**,**d**), respectively.

To further determine the temperature dependence of different water species, VT ^2^H NMR spectra were collected over the temperature range from 190 to 320 K. Figure 3 shows the dynamic-editing ^2^H spectra and peak assignment of CTS-N at 190, 280 and 300 K, and Figure 4 shows the ^2^H spectra for the total and mobile components at different temperatures. Briefly, the mobility of the different components is distinguished on the basis of their quadrupolar splitting in the ^2^H spectrum. The rigid components with confined mobility have strong quadruple interactions, which lead to a broad peak; the mobile components with high mobility exhibit a narrow peak due to the motion induced averaging of quadrupolar interactions. In general, as shown in Figure 3a, at room temperature (*i.e.*, approximately 300 K), the broad peak of the rigid ^2^H component belong to –ND/–OD from chemical exchange between rigid chitosan chains and D_2_O, and the central narrow peak in the spectra of mobile components correspond to the weakly bound and free water. On the basis of the saturation recovery quadrupolar echo ^2^H spectra shown in Figure 3b, non-freezable water exhibited a high molecular mobility, even at 190 K. In addition, the rigid amorphous component at 190 K is classified as the water strongly bound to the polymer matrix in the crystalline region. Besides, the narrow peak of the highly mobile species in the center at 190 K implies the existence of flipping water undergoing 180° flips around their bisector axis, similar to that reported for some crystalline hydrates [24]. The broad peaks of the Torchia’s T_1_ filter quadrupolar echo spectrum at 190 K came from the signals of frozen D_2_O, and –OD and the –ND components in chitosan chains, both of which cannot be distinguished at this temperature. Overall, on the basis of the VT ^2^H results, four water species were observed: free water, weakly bound water, matrix water and flipping water that undergoes well-defined 180° flips in CTS-N and CTS-A.

**Figure 5:**
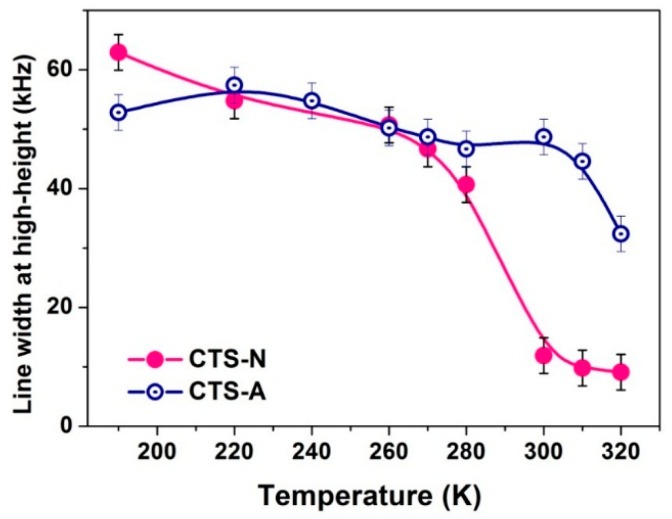
Line width at half height of the narrow components of the solid-echo spectra for CTS-N (Figure 4).

On the basis of the VT ^2^H NMR spectra shown in Figure 4, the greatest difference in the water state between the CTS-A and CTS-N samples was mostly due to the weakly bound water. The line width at half height of the central narrow peak in the spectra of mobile components (Figure 4b,d) with increasing temperature is shown in Figure 5; these results indicate that the mobility of the bound water and free water increase with increasing temperature. As shown in Figure 5, the line width of the central narrow peak for CTS-N decreased substantially after 260 K, whereas that of CTS-A changed more slowly. The line width in the ^2^H spectrum for CTS-A was around 41 kHz at room temperature, which is much larger than that of CTS-N, ~10 kHz. This result indicates that the molecular motion of a portion of the water molecules is strongly affected by the mobility of the acetic acid. This experimental result should be attributed to fact that most of the water molecules (D_2_O) in the CTS-A sample were bound to CH_3_COOH because of the stronger hydrophilic force of carboxyl groups. This behavior will be further confirmed by curves showing relative intensity of the narrow components with increasing temperature.

**Figure 6:**
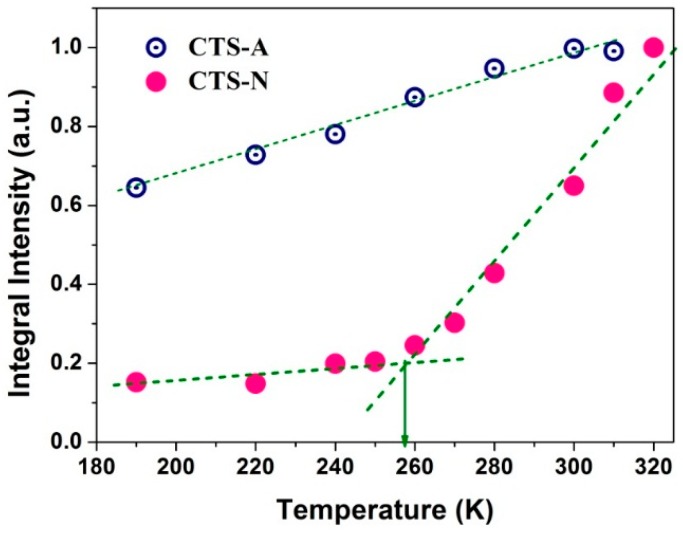
Integral signal intensity of the saturation recovery quadrupolar echo ^2^H spectra from Figure 4b,d. The onset of motion of the weakly bound matrix water is shown at 260 K.

To gain additional insight into the water dynamics, we also showed the integral intensity of the saturation recovery quadrupolar echo spectra (Figure 4b,d) with increasing the temperature as shown in Figure 6. For both CTS-A and CTS-N, the increase in the amount of mobile components with increasing temperature leads to an increase in the overall integral intensity of the saturation recovery quadrupolar echo spectra. In particular, an inflection point in the CTS-N curve was observed, which could be ascribed to the onset of the motion of the matrix water. As previously reported, the water molecules bound to the polymer matrix exhibit a glass-transition-like behavior at a temperature below 0 °C [35,36,37]. In contrast, in the case of CTS-A, the integral intensity of the mobile component increased linearly with increasing the temperature, because most of the water molecules were bound with the carboxyl groups in CH_3_COOH, which were non-freezable, even at 190 K. Few water molecules were bound to the polymer matrix, and thus no inflection point was observed.

### 3.3. Effect of Stretching on the Hydrated and Neutralized Chitosan by ^2^H Experiments

**Figure 7:**
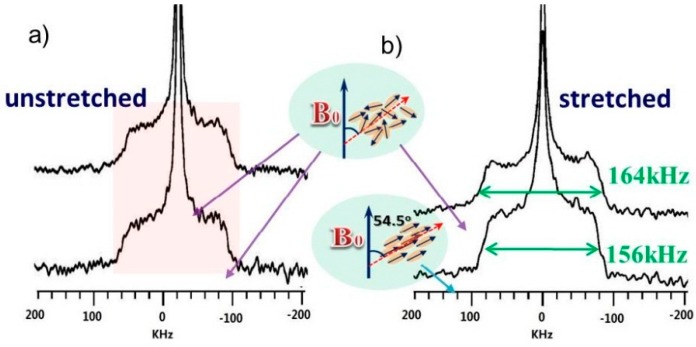
^2^H NMR spectra of unstretched (**a**) and stretched (**b**) CTS-N sample. The top spectra correspond to the randomly oriented powder samples, while the bottom spectra correspond to sample placed along the magic angle.

Solid-state ^2^H NMR is an ideal probe for investigating preferential orientation distributions [32,38,39], as the quadrupolar splitting is sensitive to the orientation of O–D/N–D bond relative to the magnetic field, as shown in Equation (1). In general, the distribution of the spectral intensity across the ^2^H NMR line shape is directly correlated to the orientation distribution of the corresponding O–D/N–D bonds. For example, a randomly oriented powder sample gives rise to a characteristic powder line shape with a spherical distribution. However, if the O–D/N–D bonds were mostly along the magic angle (*i.e.*, 54.7°), and the quadrupolar splitting will be largely reduced. The ^2^H NMR spectra of unstretched and stretched CTS-N obtained with random or oriented placement along the magic angle are shown in Figure 7. As is clearly shown in Figure 7b, the quadrupolar splitting of the ^2^H spectrum of the stretched CTS-N sample become smaller when the stretched sample was packed in the rotor along the magic angle, in comparison to that packed randomly, indicating that the polymer chains became oriented after stretching. As a control experiment, the ^2^H spectra of unstretched CTS-N were also acquired in two ways. Not surprisingly, the ^2^H spectra of unstretched CTS-N were basically the same whether the sample was packed randomly or along the magic angle. Therefore, a small amount of water could make some chitosan chains become more flexible, which would dissipate the stress in the materials and enhance the toughness. On the basis of these NMR results, the appropriate content of water bound with biopolymer chains is beneficial for enhancing the toughness of biomaterials.

## 4. Conclusions

The dynamics and different states of water in hydrated chitosan were investigated using dynamic-editing VT ^2^H solid-state NMR spectroscopy. Four distinct types of water in hydrated chitosan were detected in the chitosan samples: (1) non-freezable, rigid and strongly bound water in the crystalline domain; (2) highly mobile water that is weakly bound with polymer chains and exhibits isotropic motion, even below 260 K; (3) flipping water that undergoes well-defined 180° flips at 190 K; and (4) free water. In the case of neutralized and hydrated chitosan (CTS-N), the onset of motion of weakly bound water molecules at lower temperatures was revealed using ^2^H NMR spectroscopy, which is absent in the CTS-A sample due to the strong binding of water and carboxyl groups in CH_3_COOH molecules; these water molecules were not crystalline, even below 260 K. Furthermore, the ^2^H spectra of stretched and unstretched CTS-N samples suggested that the polymer chains became oriented after stretching. In summary, the water dynamics in chitosan films were investigated in detail by VT ^2^H spectra, which could play an important role in enhancing the mechanical properties of CTS-N sample.

## Figures and Tables

**Table 1:** Tensile properties of CTS-A and CTS-N samples.

Samples	Young’s modulus (GPa)	Yield stress (MPa)	Break strength (MPa)	Strain of break % (Elongation)
CTS-A	2.86 ± 0.05	–	84.3 ± 2.5	6.1 ± 0.8
CTS-N	2.43 ± 0.08	81.4 ± 1.6	98.7 ± 1.6	35.8 ± 5.3

## Supplementary Materials

Supplementary materials can be found at www.mdpi.com/2073-4360/8/4/149/s1.
