# Supplementary material for: 2H Solid-State NMR Analysis of the Dynamics and Organization of Water in Hydrated Chitosan
Source: Polymers (Basel). 2016 Apr 19;8(4):149. doi: 10.3390/polym8040149 (PMC6431985; doi:10.3390/polym8040149)
Supplement: Supplementary file 1 [file polymers-08-00149-s001.pdf]

Supplementary Materials: ^{2}H Solid-State NMR Analysis of the Dynamics and Organization of Water in Hydrated Chitosan

Fenfen Wang, Rongchun Zhang, Tiehong Chen and Pingchuan Sun

1. Tensile Tests for CTS Films with Different Water Contents

The tensile stress-strain curves of CTS samples with different water content were shown in Figure S1, and the relative data of tensile properties were listed in Table S1. Since the non-neutralized chitosan films would break to pieces, we can only change the water content of CTS-N. For CTS-N, the mechanical strength was decreased, while the elongation was increased with the water content increased. The chitosan chains should be plasticized by water molecules, thus the chains mobility increased which resulted in the increased toughness. The increase of the toughness will usually cause the loss of strength. However, the CTS-N with 8% water content has both excellent Young' s Modulus and toughness, while CTS-A with the same water content shows a brittle fracture. The water species and organization should give a contribution to the toughness of CTS-N, which were different from that of CTS-A. So, the main research objects were CTS-A and CTS-N with 8% water content in the main text of this paper.

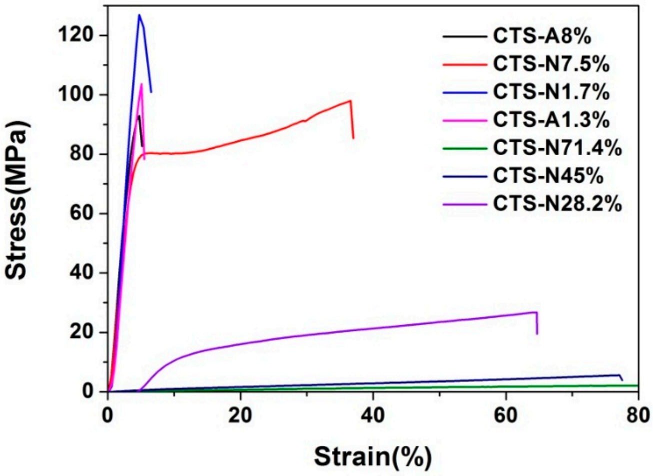

Figure S1. Tensile tests for CTS films with different water contents. Water contents were determined by TGA as list in upper right.

Table S1. Tensile properties of CTS-A and CTS-N samples with different water content.

Samples	Young' s modulus (GPa)	Yield stress (MPa)	Break strength (MPa)	Strain of break (%) (Elongation)
CTS-A(1.3%)	2.9 ± 0.2	——	103.6 ± 3.2	5.5 ± 0.6
CTS-A(8%)	2.8 ± 0.05	——	84.3 ± 2.5	6.1 ± 0.8
CTS-N(1.7%)	3.3 ± 0.2	——	126.2 ± 2.3	4.8 ± 0.5
CTS-N(8%)	2.43 ± 0.08	81.4 ± 1.6	98.7 ± 1.6	41.4 ± 5.3
CTS-N(28.2%)	0.06 ± 0.02	——	25.3 ± 3.6	62.8 ± 5.5
CTS-N(45%)	——	——	5.5 ± 1.2	78.3 ± 5.2
CTS-N(71.4%)	——	——	5 ± 1.2	200 ± 5

2. Thermo-Gravimetric Analysis (TGA) of CTS Samples

In order to determine the water content in chitosan films, TGA analysis was employed here, the results were shown in Figure S2. The resulting water content is approximately 8 wt % in both CTS-A and CTS-N sample. TGA measurements were carried out on a TGA 500 (TA Instruments). The moisture content was measured by heating the samples from 25 to 800 °C under nitrogen atmosphere using a ramp of 5 °C/min. The percentage of moisture was calculated from the weight lost at the end of 200 °C.

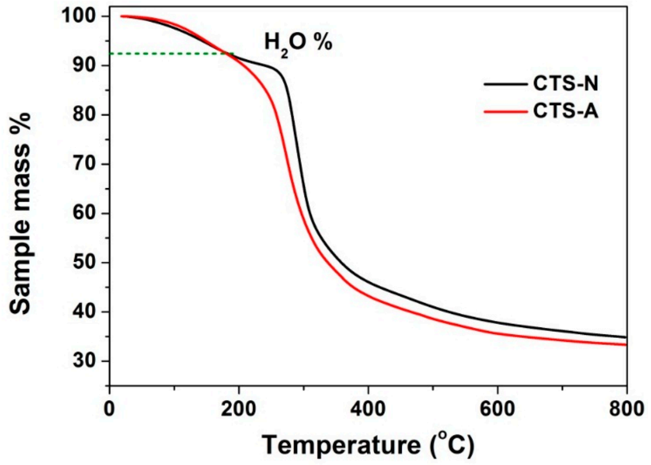

Figure S2. TGA curves of CTS-A and CTS-N.

3. XRD Experiments

The XRD diffractograms were shown in Figure S3. The degree of crystallinity (Xc) was estimated by visual examination. The broad bump shown by the blue line belongs to the amorphous phase of chitosan, and the other peaks are attributed to the crystalline phase. The crystallinity (Xc) was calculated by the following formula:

$$Xc = I_{crystal}/(I_{crystal} + I_{amorphous}) \times 100\% \quad \text{(S1)}$$

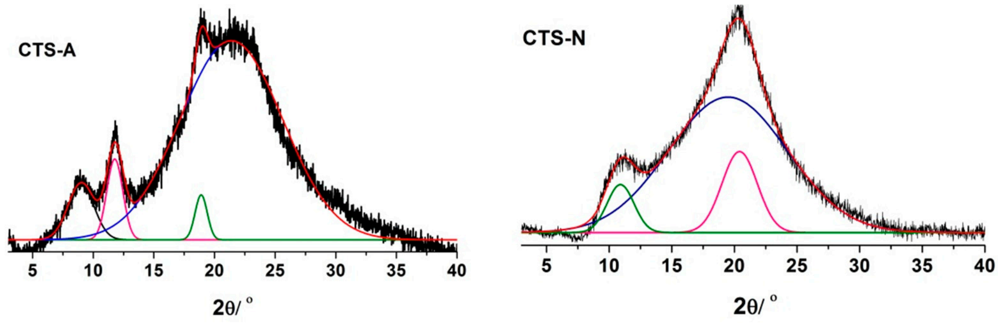

Figure S3. XRD diffractograms of CTS-A and CTS-N.

4. ^{2}H NMR Experiments for Chitosan Films

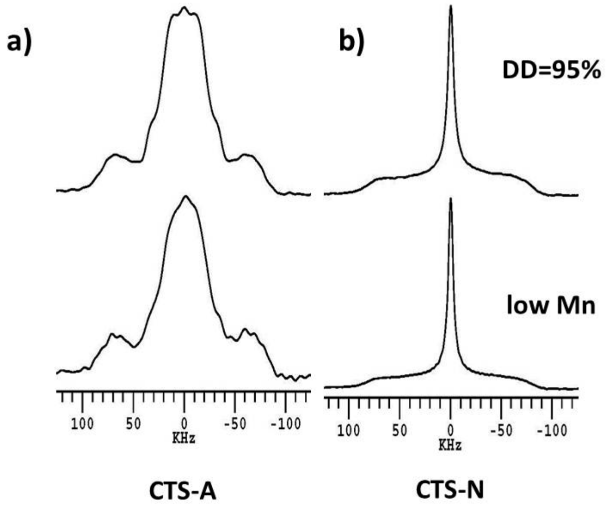

Figure S4. ^{2}H NMR for chitosan samples (**a**) before neutralized; and (**b**) after neutralized. The above is spectra of chitosan with high degree of deacetylation, approximately 95%; the bottom is spectra of chitosan with low molecular weight.

© 2016 by the authors; licensee MDPI, Basel, Switzerland. This article is an open access article distributed under the terms and conditions of the Creative Commons Attribution (CC-BY) license (http://creativecommons.org/licenses/by/4.0/).
